# Retracted: Induction of Heat Shock Protein Expression in Cervical Epithelial Cells by Human Semen

**DOI:** 10.1155/2017/7464838

**Published:** 2017-11-13

**Authors:** Infectious Diseases in Obstetrics and Gynecology

Infectious Diseases in Obstetrics and Gynecology has retracted the article titled “Induction of Heat Shock Protein Expression in Cervical Epithelial Cells by Human Semen” [1]. The article was found to report the same results, without citation, as the following published article: J. Jeremias, S. S. David, M. Toth, S. S. Witkin, “Induction of messenger RNA for the 70 kDa heat shock protein in HeLa cells and the human endocervix following exposure to semen: implications for antisperm antibody production and susceptibility to sexually transmitted infections,” Human Reproduction (1997) 12 (9): 1915–1919. DOI: https://doi.org/10.1093/humrep/12.9.1915 [2]. The last author does not approve of retraction and the other authors could not be contacted.
